# Safety Assessment of the Auto Manipulation Device for Acupuncture in Sprague-Dawley Rats: Preclinical Evaluation of the Prototype

**DOI:** 10.1155/2018/5708393

**Published:** 2018-08-06

**Authors:** Geng-Hao Liu, Meng-Yen Tsai, Gwo-Jyh Chang, Chao-Min Wu, Sheng-Kai Lin, Yu-Sheng Chen, Tzung-Yan Lee

**Affiliations:** ^1^Division of Acupuncture and Moxibustion, Department of Traditional Chinese Medicine, Chang Gung Memorial Hospital, Linkou, Taiwan; ^2^Graduate Institute of Clinical Medical Sciences, College of Medicine, Chang Gung University, Taoyuan, Taiwan; ^3^School of Traditional Chinese Medicine, Chang Gung University, Taoyuan, Taiwan; ^4^Sleep Center, Chang Gung Memorial Hospital, Taoyuan, Taiwan; ^5^Department of Electrical Engineering, National Central University, Jhongli, Taiwan; ^6^Delta Electronics, Inc., Taoyuan, Taiwan; ^7^Graduate Institute of Traditional Chinese Medicine, College of Medicine, Chang Gung University, Taoyuan, Taiwan; ^8^Department of Traditional Chinese Medicine, Chang Gung Memorial Hospital, Keelung, Taiwan

## Abstract

**Background:**

The Auto Manipulation Device for Acupuncture (AMDA) is designed for providing stable, quantified effects and higher frequency when doing lifting and thrusting manipulation. The purpose of this study is to investigate the safety of manipulation by AMDA in different frequency and duration in healthy rats.

**Methods:**

The study was divided into two parts: single intervention and once a day for a week. 12 rats and 15 rats were randomly allocated to different groups: Control (needle insertion only), AMDA (2Hz/10Mins), AMDA (2Hz/20Mins), AMDA (20Hz/10Mins), and AMDA (20Hz/20Mins) for single and repeated interventions. Real-time physiological functions, laboratory data, and the bilateral muscle tissue of acupoint (ST 36) were obtained after the intervention.

**Results:**

We found neither real-time physiological functions nor laboratory data differences between control group and AMDA groups in both parts. In the muscle tissue samples, the slight damage had been observed in the AMDA group with a frequency of 2 Hz for 20 minutes after once intervention, and the repeated session groups noted more obvious tissue damage with fibrotic change. Although the period was shorter, higher frequency manipulation caused more damage that fibroblast nuclei became more slender and obvious. However, no significant adverse effect was noted such as crippled and molting in the whole process.

**Conclusion:**

Our study suggested that the safety issue of AMDA operation in rats is feasible because there was no difference between control group and AMDA groups among real-time physiological functions and laboratory data. However, manipulation with higher frequency should be more preserved.

## 1. Introduction

Acupuncture is one of the most frequently requested complementary therapies [1]. Various disorders can effectively be cured by inserting long, fine needles into specific “acupuncture points” (acupoints) on the skin of the patient's body. Besides China, acupuncture has spread to over 160 countries and regions. The World Health Organization recommends the use of acupuncture treatment for 43 diseases [2]. However, acupuncture needle manipulation is one of the most fundamental yet widely variable components of acupuncture treatments [3].

Because of the variability forms of manipulation and individual difference of acupuncturists, there exist artificial errors that make scientific studies more difficult to quantize. For providing stable and quantified effects, many researchers provide devices to monitor the frequency and amplitude of manipulation [4]. The Auto Manipulation Device for Acupuncture (AMDA) is designed for providing stable, quantified effects, and higher frequency when doing lifting and thrusting manipulation. The preliminary results have demonstrated the developed AMDA and its plausibility in the clinical application of acupuncture in simulated tissues [5].

The purpose of this study is to investigate the new method of manual manipulation (MA) by AMDA and the safety of different frequency and duration after acupuncture intervention in healthy rats.

## 2. Material and Methods

**Figure 1:**
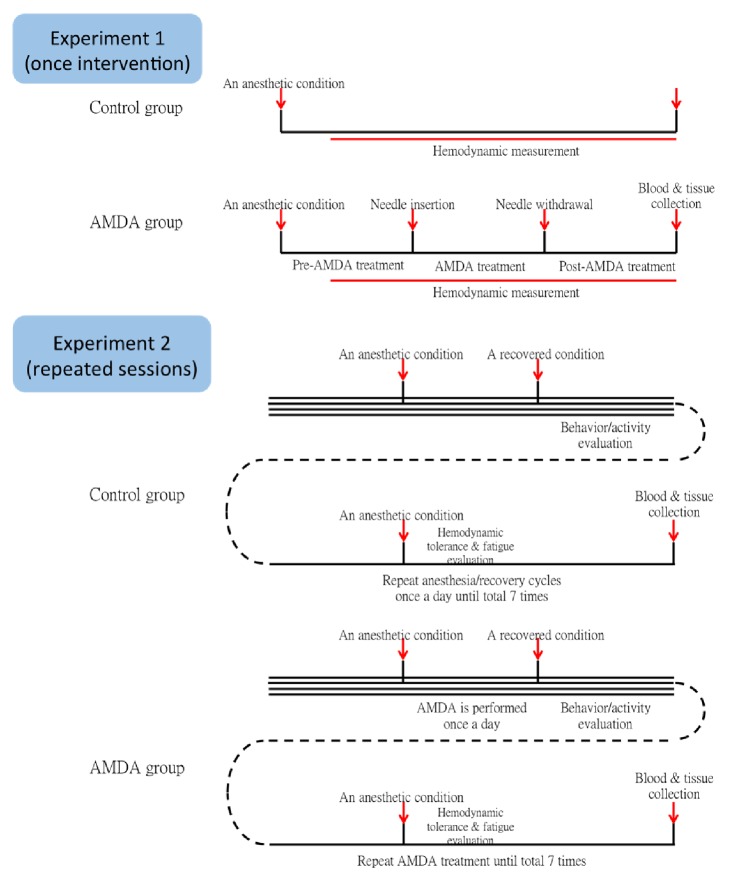
Protocol of experiment 1 (once intervention) and experiment 2 (repeated sessions).

The study was divided into two parts: single intervention and once a day for a week (the detailed protocol was shown in Figure 1). Manual lifting-thrusting acupuncture was given with Auto Manipulation Device for Acupuncture (AMDA, prototype).

### 2.1. Animal Preparation and Recording Procedures

27 healthy male Sprague-Dawley rats weighing 280±50 g and aged 6-8 weeks were provided by the Bio LASCO animal centre. The animals were maintained in a controlled environment (22±2°C and 50±5% humidity) and under a 12 h/12h light/dark cycle with free access to food and water. This study was performed in accordance with the Guidance Suggestions for the Care and Use of Laboratory Animals of the Animals in Science Regulation Unit of UK. All experimental procedures were approved by the Chang Gung University Institutional Animal Care and Use Committee (IACUC Approval no. CGU15-088) and were conducted in a manner that minimized the number of animals used and the number of procedures per animal.

In experiment 1 (once intervention), 12 rats were randomly allocated to different groups: Control (needle insertion only, AMDA 0 Hz/20Mins, n=4), AMDA^ls^ (2Hz/10Mins), AMDA^ll^ (2Hz/20Mins), AMDA^hs^ (20Hz/10Mins), and AMDA^hl^ (20Hz/20Mins) (n=2 each AMDA group). Real-time physiological functions, including heart rate, systolic blood pressure, mean arterial pressure, diastolic blood pressure, are measured using the tail-cuff method, recorded before the manual acupuncture with AMDA intervention and every five minutes during the course. Then, blood samples, including hepatic, renal function (AST/ALT, BUN/Cr), electrolytes (Na/K), and hemogram (CBC/DC), were also collected after sacrifice. Third, the bilateral muscle of ST36 acupoint was harvested and the histological sections were stained with hematoxylin and eosin (H&E) and were observed under a light microscope (40X, 100X) after the intervention.

In experiment 2 (repeated sessions), all 15 rats received daily manual ST36 acupuncture with AMDA intervention for 7 days and were randomly allocated to different groups: Control (0Hz/20Mins, n=3), AMDA^LS^ (2Hz/10Mins), AMDA^LL^ (2Hz/20Mins), AMDA^HS^ (20Hz/10Mins), and AMDA^HL^ (20Hz/20Mins) (n=3 each AMDA group). Real-time physiological functions, body weight change, the muscle histological sections, and blood samples were also collected in the seventh days.

### 2.2. Experimental Procedures

**Figure 2:**
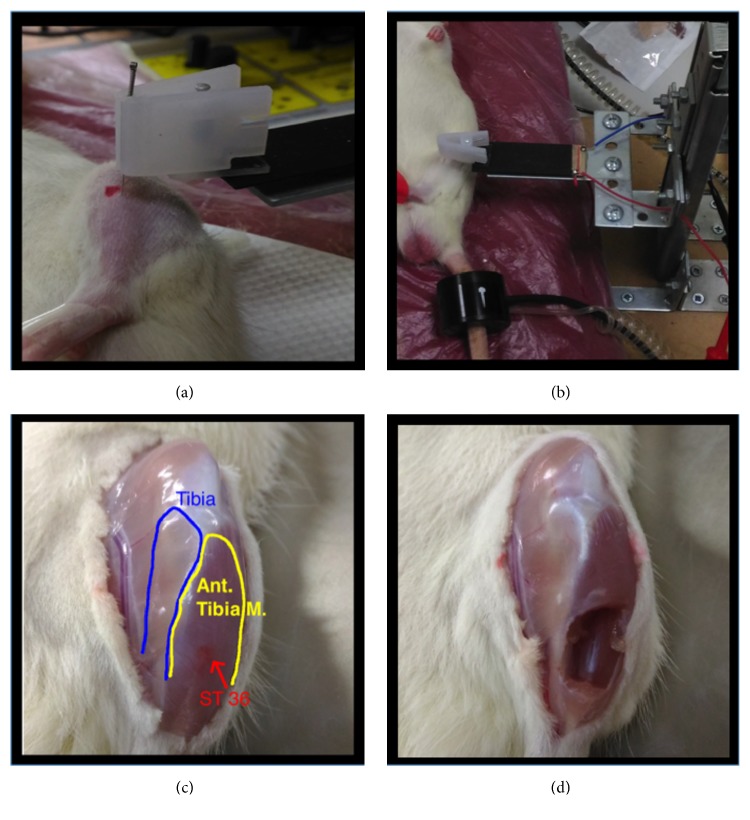
**Manual acupuncture at ST36 with AMDA and tissue sampling from ST 36. (a)** ST 36 was located 4 mm bellowed and 1-2 mm lateral to the midpoint of the knee. Manual acupuncture with AMDA** (b) **was performed after well anesthesia.** (c)** After rechecking the margin of muscle (yellow) and bone (blue), the muscle tissues of ST 36 (red) were obtained** (d).**

The rats were kept in supine position under anesthesia with 4% isoflurane inhalation and maintain the depth of anesthesia as stage III, which was assessed by pedal reflex, preserve normal body temperature using warm thermal pads. The ST36 point in the rat is located at a point 5 mm lateral and inferior to the tibial tubercle. Based on the comparative anatomical localization in rats as compared with that in human, selected points (the location of acupoints is shown in Figure 2) [6, 7].

The region of acupoint was shaved and disinfected; and then a sterilized single-use stainless steel needle measuring 0.27 mm ×13 mm (0.27 mm in diameter and 13 mm in length; Ching-Ming Medical Co., Ltd., Taiwan) was placed on the left side ST36 by a single experienced acupuncturist. The insertion depth was about 6 mm. After the* de qi* sensation, the AMDA was connected to the handle of the acupuncture needle. Afterward, adjust the device to the specific frequency and start the lifting-thrusting manipulation for each group.

In experiment 2, acupuncture was repeated for 7 days. All rats were sacrificed by decapitation, and tissue samples were collected and analyzed [8].

The procedure was carried out in accordance with the IACUC Guidelines.

### 2.3. Statistical Analysis

Statistical analysis was conducted using IBM SPSS Statistics 21.0 Software (IBM Corp. Released 2012. IBM SPSS Statistics for Windows, Version 21.0. Armonk, NY: IBM Corp.). All data were analyzed using Kruskal-Wallis ANOVA test and presented as mean±SD of the mean (SEM). Significance was considered when p<0.05.

## 3. Results

### 3.1. Experiment 1 (Once Intervention)

In experiment 1 (once intervention), real-time physiological functions before the intervention were collected and showed no significant difference in each group (all P>0.05). After the intervention, we found neither real-time physiological functions nor blood samples differences between control group and AMDA groups (Table 1).

**Figure 3:**
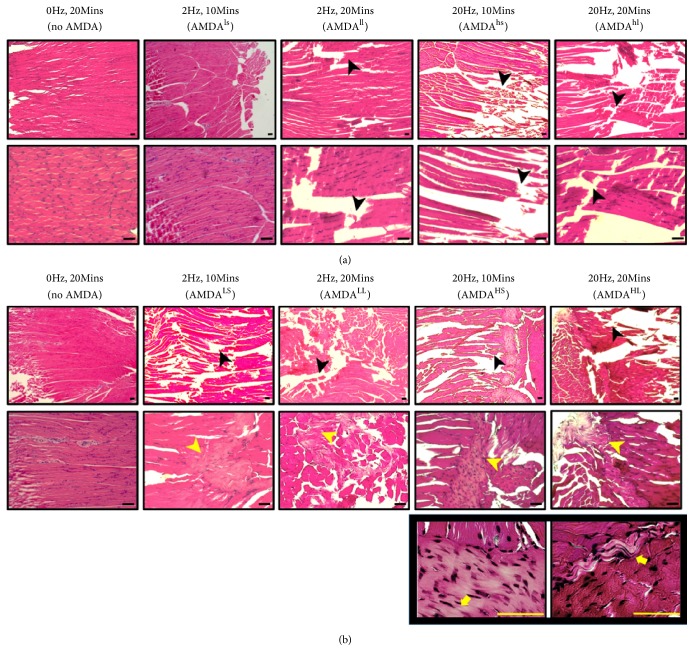
Once intervention (a) and repeated sessions (b) of AMDA effect on muscle tissue at acupoint ST36 in different needle frequency and retention time. The bar of proportional scale is 100 um. AMDA with higher frequency (20Hz) make clearly visible tissue tear (black arrowhead). Regardless of the needle frequency in repeated sessions, fibrotic change (yellow arrowhead) was noted. As the needle retention time was extended to 20 minutes, the fibroblast nuclei (yellow arrow) are also more slender and obvious. (a) Once intervention. ls, low frequency (2Hz) and short duration (10mins); ll, low frequency (2Hz) and long duration (20mins); hs, high frequency (2Hz) and short duration (10mins); hl, high frequency (2Hz) and long duration (20mins). (b) Repeated sessions. LS, low frequency (2Hz) and short duration (10mins); LL, low frequency (2Hz) and long duration (20mins); HS, high frequency (2Hz) and short duration (10mins); HL, high frequency (2Hz) and long duration (20mins).

In the muscle tissue samples, the slight damage had been observed in the AMDA group with a frequency of 2 Hz for 20 minutes (Figure 3(a)). As long as the period prolonged, the damage increased in an order: control (AMDA 0 Hz/20Mins) < AMDA^ls^ (2Hz/20Mins) < AMDA^hs^ (20Hz/10Mins) < AMDA^hl^ (20Hz/20Mins) (Figure 3(a)).

### 3.2. Experiment 2 (Repeated Sessions)

After 7 days of repeated acupuncture intervention, the data presented in Table 2 showed that it still had no significant change in the real-time physiological function, including heart rate, systolic blood pressure, mean arterial pressure, and diastolic blood pressure in the rats. Also, it did not affect hepatic or renal function (AST/ALT, BUN/Cr), electrolyte (Na/K), or hemogram (CBC/DC).

In addition, there was no significant body weight change between those groups (P=0.220). No significant adverse effect was noted such as crippled and molting in the whole process.

In the muscle tissue samples, the damage had been observed in all AMDA groups. In comparison to once intervention group with a frequency of 2Hz for 10 minutes, the repeated session groups noted more obvious tissue damage with fibrotic change (Figure 3(b)). Our study found that although the period was shorter, higher frequency manipulation causes more damage that fibroblast nuclei became more slender and obvious (Figure 3(b)). In conclusion, the damage increased in an order: control (AMDA 0 Hz/20Mins) < AMDA^LS^ (2Hz/10Mins) < AMDA^LL^ (2Hz/20Mins) < AMDA^HS^ (20Hz/10Mins) < AMDA^HL^ (20Hz/20Mins).

## 4. Discussion

The main purpose addressed by this study was whether AMDA was available for providing a stable and safe new method acupuncture manipulation with different frequency and duration in rats. As most patients receive more than one acupuncture therapy in the treatment course, this study was conducted in two phases for investigating short-term and repeated effects. We hypothesized that MA manipulation by AMDA may affect the rats in three ways: real-time physiological functions, laboratory finding, and tissue damage. It follows that neither real-time physiological functions nor laboratory blood test differences between control group and AMDA groups in both time courses. Moreover, there was no significant adverse effect noted during the course. The results presented here reveal the safety issue of AMDA operation in rats is feasible.

However, according to the histologic review, a  single  AMDA  operation with 20 Hz will have a slight damage to muscle tissue. After daily manipulation with AMDA, regardless of the needle frequency, muscle tissue over acupoint (ST36) became fibrosis. Moreover, AMDA with higher frequency (20Hz) make clearly visible tissue tear. As the needle retention time was extended to 20 minutes, the fibroblast nuclei are also more slender and obvious.

For fibroblasts and endothelial cells, focal adhesions form mechanical links between extracellular collagen matrix and intracellular cytoskeleton. It was evident that the needle movement was an effective mechanical stimulus leading to tissue displacement [9]. Tissue tension is likely sensed by fibroblast by their adhesion to collagen fibers [10, 11]. Langevin observed that mechanical coupling between the needle and connective tissue with a winding of tissue around the needle during needle manipulation transmits a mechanical signal to connective tissue cells via mechanotransduction [9, 12].

We knew that the therapeutic effectiveness of acupuncture could be influenced by multiple factors. Basic science experiments, mostly in animals and healthy human subjects, show that acupuncture needling has demonstrable physiological effects that are dependent on needling parameters, including needle insertion depth, type, amplitude, and frequency of needle stimulation [13]. Between therapeutic effectiveness of acupuncture and connective tissue damage, the control of parameters of manipulation was quite important. In this study, we can deduce that the most important factor cause tissue damage is the frequency of manipulation, and then it is the time of needle retention and repeated times of manipulation.

Another study that evaluates the variable frequencies of manual acupuncture at ST36 in rats with atropine-induced inhibition of gastric motility also found that twirling manipulations with frequencies of 1, 2, and 3 Hz had better therapeutic effects than a frequency of 4 Hz on the recovery of the gastric motility amplitude [14]. Therefore, the frequency of manual acupuncture influences not only the therapeutic effects but the safety of acupuncture intervention.

On the other hand, electroacupuncture at ST36 increases the concentration and reorganization of collagen in the rat model of tendon healing [15]. The subtle differences in acupuncture needle manipulation techniques can affect cellular responses in mouse subcutaneous connective tissue [16]. Further studies will be needed to determine whether those manipulations are related to therapeutic responses.

Now, modern imaging and cell biology techniques have been employed to study the nature of acupuncture and many researchers build many models to explain acupuncture such as mechanistic function, neural response, or electrical response. For example, earlier studies have shown that rotation of an inserted acupuncture needle stretches nearby connective tissue by pulling collagen fibers from the periphery toward the needle [11, 17]. On the other hand, current study found that the acoustic shear wave, being a mechanical energy, is capable of mechanotransduction, stimulating cytosolic Ca2+ rise in both excitable and nonexcitable cells, producing Ca2+ oscillations and memory, and giving rise to in vivo calcium fluorescence and endorphin release into blood plasma in mice [18]. However, there were some current studies which suggest that the initial action of acupuncture appears to be mechanical and not neural or electrical. The mechanism of acupuncture still needs further evidence to be proved.

As for the manipulation effect for fibroblasts, previous studies have shown that both acupuncture needle rotation and simple tissue stretching cause fibroblasts to increase their cross-sectional area, as their cell bodies expand and spread out [11, 19]. The fibroblast responsiveness along a plane of connective tissue could be the source of purines that led to adenosine-mediated acupuncture analgesia some distance away from the needle [20]. The response of fibroblasts to acupuncture still needs further study to distinguish the benefit and the adverse effect of a high frequency intervention.

However, the size proportion of the needle to the body of a rat was different from that of a human. Thus, the muscle tissue damage may be overrated in the animal model. Even though our study only focuses on rats, the damage of repeated needle intervention still was an important issue. Skin changes such as localized lipoatrophy and hypertrophic scar have been reported in some review articles, especially during a relatively long treatment period [21]. In some case reports, epithelioid granuloma, pseudolymphoma, and scars at needling sites were also mentioned [22].

Guidelines of World Health Organization on basic training and safety in acupuncture have proposed the safety in acupuncture, including prevention of infection, contraindications, management of accidents, and untoward reactions [23]. We suggested avoiding high frequency and repeated manipulation, based on our result of muscle pathology and clinic observation of patients with acupoint-fibrosis. At the same time, try to avoid the same acupoint in the treatment course for decreasing the stimulation of same muscle tissue and the incidence of muscle tissue fibrosis.

One advantage of the study is that we try to provide a new and safe method for acupuncture study to solve the variability forms of manipulation and individual difference of acupuncturists, which is another way to reduce artificial errors and quantified the effects. The limit of our study includes the related small amount sample and may need further study for evaluation the fibrosis tissue and the differences between manipulations. On the other hand, even though the experienced acupuncturist performed the needle manipulation to the* de qi* sensation first, the followed AMDA could not sense and maintain it in the whole process. Another question that may be asked is whether the finding from rats is applicable to humans.

## 5. Conclusions

Our study suggested that the safety issue of AMDA operation in rats is feasible because there was no difference between control group and AMDA groups among real-time physiological functions and laboratory sample test in both intervention courses. However, lifting-thrusting manipulation with higher frequency should be more preserved, especially in patients that need more often acupuncture intervention, such as chronic arthritis, sciatica, and cerebrovascular disease. Further studies will be needed to investigate the potential of AMDA.

## Figures and Tables

**Table 1:** Baseline vital signs, hemogram, and biochemistry laboratory data in single intervention.

Baseline vital signs	Control group (n=4)	AMDA group (n=8)	P
HR (bpm)	377.8±15.9	355.9±16.8	.056
SBP (mmHg)	89.0±4.5	85.6±6.5	.379
MBP (mmHg)	70.3±4.6	63.1±7.3	.109
DBP (mmHg)	61.0±5.0	52.0±9.5	.110

Vital signs	Control group (n=4)	AMDA group (n=8)	P

HR (bpm)	431.3±43.4	423.4±40.1	.734
SBP (mmHg)	103.5±20.6	101.4±18.5	.932
MBP (mmHg)	80.5±9.7	80.1±12.9	.865
DBP (mmHg)	69±4.8	69.8±10.3	1.000

Hemogram			
WBC (1000/uL)	5.25±3.78	5.79±3.32	.610
RBC (million/uL)	5.013±1.513	5.28±1.496	.610
Hb (g/dL)	10.63±3.43	11.19±3.25	.552
Hct (%)	33.65±11.01	35.83±10.34	.396
MCV (fL)	66.7±2.28	67.81±2.68	.497
MCH (pg/Cell)	21.08±0.67	21.18±0.64	.495
MCHC (gHb/dL)	31.6±0.22	31.23±0.73	.200
Plt (1000/uL)	436.5±494.4	470.3±420.6	.552
Seg (%)	21.98±12.26	15.13±6.04	.308
Lym (%)	74.3±14.29	82.56±6.1	.396
Mono (%)	3.45±2.34	1.75±0.99	.234
Eosin (%)	0.1±0.2	0.38±0.43	.096
Baso (%)	0.18±0.21	0.19±0.15	.930

Biochemistry laboratory data			
BUN (mg/dL)	11.95±3.72	11.24±2.33	.609
Cr (mg/dL)	0.27±0.062	0.244±0.041	.670
Na (mEq/L)	146±0.8	146.1±1.8	1.000
K (mEq/L)	5.93±0.57	6.59±0.29	.061
AST (U/L)	112.3±5.3	111.3±7.8	.932
ALT (U/L)	44.5±4.2	49.6±7.5	.147

HR, heart rate; SBP, systolic blood pressure; MBP, mean blood pressure; DBP, diastolic blood pressure. WBC, white blood cell; RBC, red blood cell; Hb, hemoglobin; Hct, hematocrit; MCV, mean corpuscular volume; MCH, mean corpuscular hemoglobin; MCHC, mean corpuscular hemoglobin concentration; Plt, platelet; Seg, segment; Lym, lymphocyte; Mono, monocyte; Eosin, eosinophil; Baso, basophil. BUN, blood urea nitrogen; Cr, Creatinine; Na, sodium; K, potassium; AST, aspartate aminotransferase; ALT, alanine aminotransferase.

Mean ± standard deviation was presented for vital signs (HR, SBP, MBP, and DBP), hemogram (complete blood count and differential count), and biochemistry laboratory data (BUN/Cr, Na/K, and AST/ALT). No significant difference between the 2 groups was found at baseline and once intervention study.

**Table 2:** Vital signs, hemogram, and biochemistry laboratory data in repeated sessions study.

	Control group (n=3)	AMDA group (n=12)	P
Rat body weight gain (%)	12.85±2.25	12.69±2.65	.942

Vital signs			
HR (bpm)	411.3±17.4	382.7±29.7	.149
SBP (mmHg)	84.7±11.2	87.1±9.8	.771
MBP (mmHg)	68.3±13	67.3±10.6	1.000
DBP (mmHg)	59.3±14.2	57.6±11.8	.828

Hemogram			
WBC (1000/uL)	7.4±2.96	8.43±4.09	.613
RBC (million/uL)	6.357±1.384	6.801±0.698	.773
Hb (g/dL)	13.47±2.8	14.21±1.3	.772
Hct (%)	41.83±8.82	44.48±3.89	.773
MCV (fL)	65.87±0.81	65.58±3.11	.563
MCH (pg/Cell)	21.2±0.26	20.93±0.89	.347
MCHC (gHb/dL)	32.23±0.21	31.94±0.36	.128
Plt (1000/uL)	516.7±373.2	523.7±322.2	.885
Seg (%)	16.33±2.25	16±7.41	.248
Lym (%)	81.5±2.43	81.45±7.45	.278
Mono (%)	1.4±0.1	1.53±0.93	.884
Eosin (%)	0.5±0.2	0.76±0.6	.563
Baso (%)	0.27±0.06	0.26±0.12	.939

Biochemistry laboratory data			
BUN (mg/dL)	15.2±3.87	14.3±3.43	.773
Cr (mg/dL)	0.213±0.015	0.23±0.045	.716
Na (mEq/L)	145.3±1.5	146.8±2.2	.210
K (mEq/L)	6.57±0.12	6.93±0.61	.346
AST (U/L)	121.7±16.3	125.8±23.7	.885
ALT (U/L)	50.7±2.3	54.1±8.1	.469

Mean ± standard deviation was presented for the percentage of rat body weight gain, vital signs (HR, SBP, MBP, and DBP), hemogram (complete blood count and differential count), and biochemistry laboratory data (BUN/Cr, Na/K, and AST/ALT). No significant difference between the 2 groups was found at chronic AMDA study.

## Data Availability

The data used to support the findings of this study are available from the corresponding author upon request.

## Supplementary Materials

Supplementary Materials The low and high frequency (2/20 Hz) manipulation of the AMDA prototype have been recorded as a video file and as a supplementary document.
